# Relationships Between Childhood Bullying/Domestic Violence Experience and Insomnia among Employees in Japan

**DOI:** 10.12688/f1000research.129340.2

**Published:** 2023-07-14

**Authors:** Kei Muroi, Mami Ishitsuka, Daisuke Hori, Shotaro Doki, Tsukasa Takahashi, Tomohiko Ikeda, Tamaki Saito, Sasahara Shinichiro, Ichiyo Matsuzaki

**Affiliations:** 1 Graduate School of Comprehensive Human Sciences, University of Tsukuba, Tsukuba, Ibaraki, 305-8575, Japan; 2 Faculty of Medicine, University of Tsukuba, Tsukuba, Ibaraki, 305-8575, Japan; 3 International Institute for Integrative Sleep Medicine, University of Tsukuba, Tsukuba, Ibaraki, 305-8575, Japan

**Keywords:** Adverse Childhood Experiences, Sleep Disturbance, Athens Insomnia Scale, Cross-Sectional, Employees

## Abstract

Objective: Traumatic childhood experiences such as domestic violence and bullying have been reported to be associated with insomnia in adulthood. However, little evidence is available for the long-term effects of childhood adversity on workers’ insomnia worldwide. Our objective was to examine whether childhood experiences of bullying and domestic violence are associated with insomnia in workers in adulthood.

Methods: We used survey data from a cross-sectional study of the Tsukuba Science City Network in Tsukuba City, Japan. Workers aged 20 to 65 years (4509 men and 2666 women) were targeted. Binomial logistic regression analysis was performed with the Athens Insomnia Scale as the objective variable.

Results: Binomial logistic regression analysis showed that childhood bullying and domestic violence experience of childhood bullying and domestic violence were associated with insomnia. As for the experience of domestic violence, the higher the time of experience, the higher the odds ratio of insomnia.

Conclusions: It may be useful to focus on childhood traumatic experiences regarding insomnia in workers. In the future, objective sleep time and sleep efficiency should be evaluated using an activity meter and other methods to verify the effects of bullying and domestic violence experiences.

## Introduction

Insomnia is an important public health problem, affecting about 30% of the worldwide population ( Bhaskar et al., 2016). Insomnia increases the risk of mental disorders, suicide, and chronic health conditions such as obesity, diabetes, and cardiovascular disease ( Sofi et al., 2012; McCall & Black, 2013; Biddle et al., 2018; Cai et al., 2018; Nishitani et al., 2018; Zhang et al., 2019). In Japan, Itani and colleagues conducted a nationwide interview survey and reported that 12.2% of men and 14.6% of women in the country had insomnia symptoms, eg, difficulty initiating sleep, difficulty maintaining sleep, or early morning waking ( Itani et al., 2016). The OECD Health Statistics 2019 survey revealed that Japan has the shortest average sleep time among developed countries. Thus, insomnia is one of the most important challenges in Japan ( OECD, 2020).

Socioeconomic factors such as low income, low education, divorce, and bereavement have been reported to be associated with insomnia ( Lallukka et al., 2012; Kawata et al., 2019). Also, lifestyle factors such as smoking, lack of exercise, and chronic diseases have been reported to be risks for insomnia ( Brook et al., 2015; Koyanagi et al., 2015; Kelley & Kelley, 2017). Job stress is considered an occupational risk factor for insomnia, with the evidence showing a suspected relationship between job stress and sleep problems including insomnia ( Utsugi et al., 2005; Kim et al., 2011).

Adverse Childhood Experiences (ACEs), defined as physical, psychological, or sexual abuse, bullying victimization, or family dysfunction experienced before age 18, are a public health problem ( Anda et al., 2006; Brown et al., 2009; Chapman et al., 2011; McKay et al., 2021). People who experience ACEs are mentally and psychologically vulnerable. It has been suggested that ACEs is a risk factor for sleep disturbance in adulthood ( Kajeepeta et al., 2015; Brindle et al., 2018; Sullivan et al., 2019).

Little evidence is available for the long-term effects of childhood adversity on workers’ insomnia worldwide. Examining the effects of childhood bullying and domestic violence (DV) insomnia in workers can shed light on new causes of worker insomnia that have one unreported until now in addition to job stress.

In the present study, we investigated whether experiences of bullying/DV are related to insomnia among employees in Japan.

## Methods

### Design and participants

We conducted a cross-sectional study using data obtained from a survey of the Tsukuba Science City Network in Tsukuba, a city situated about 70 km northeast of Tokyo. The Tsukuba Science City Network is an organization of the city’s research and academic institutes that aims to promote cooperation among its member institutes ( MEXT, n.d.). The Tsukuba Science City Network conducts a mental health survey every 5 years of the researchers and engineers from the member institutes in Tsukuba city. From February to March 2017, we conducted the survey via an anonymous web questionnaire entitled *The 7th Life Environment and Workplace Stress Survey.* The total number of subjects in this study was 19,481 workers at 53 research institutes in Tsukuba City, all of whom were affiliated with the Tsukuba Science City Network. These workers were contacted via e-mail through the general affairs department of each institution and directed to a self-administered questionnaire form via a URL described in the e-mail. Participants were given the option to choose either the Japanese or English version of the questionnaire. All participants remained anonymous, and no data regarding their institute affiliation was obtained. The results of this survey have been published on the Tsukuba Science City Network website, and several studies using this survey have been reported ( Hori et al., 2019; Takahashi et al., 2019; Ikeda et al., 2020). The total number of respondents was 7255, yielding a response rate of 37.2%. Sixty participants were excluded due to missing data or no response. The final sample consisted of 7175 participants (average age: 44 years; 4509 men and 2666 women) aged 20 to 65 years who answered the main response items. The detailed protocols are available at protocols.io (DOI: dx.doi.org/10.17504/protocols.io.14egn24nmg5d/v1).

### Measures

***Experiences of bullying/DV***

We collected data about experience of bullying/DV by means of an original questionnaire. The experience of bullying victimization was defined by answering “yes” to the question “Have you ever been bullied by others?” Similarly, the experience of DV victimization was defined by answering “yes” to the question “Have you ever been a victim of violence by a family member?” We observed when the experienced occurred (elementary school, junior high school, high school, or university and after). The respondents were able to select overlapping periods of time when they experienced victimization. They were classified into five groups according to the number of experiences; never experienced at all, experienced one period, experienced two periods, experienced three periods, and experienced four periods. We defined the experience from early childhood through high school years as a childhood experience and the experience from university years and after as a current experience.

Therefore, childhood bullying or DV victimization experiences were divided into four groups; never experienced at all, experienced one period, experienced two periods, and experienced three periods.

***Athens Insomnia Scale (AIS)***

The outcome variable analyzed in this study was insomnia, which was measured using the Japanese version of the AIS ( Okajima et al., 2013). This self-administered questionnaire assesses insomnia according to the 10th revision of the International Classification of Diseases criteria ( Soldatos et al., 2000; Okajima et al., 2013). The Japanese version of the AIS has been validated ( Okajima et al., 2013) and is commonly used in epidemiological studies. It consists of 8 items: the first 5 assess difficulties with sleep induction, waking during the night, early-morning waking, total sleep time, and overall sleep quality, while the remaining 3 measure the consequences of insomnia during the day, including problems with sense of well-being, overall functioning, and sleepiness. Respondents were asked to rate their experiences with these symptoms over the past month on a 4-point scale ranging from 0 (“not problematic at all”) to 3 (“extremely problematic”). The total score ranges from 0 to 24. A score of less than 4 indicates no problems, while a score of 4 to 5 suggests that consultation with a physician may be necessary (some suspicion of insomnia). A score greater than 6 indicates that consultation with a physician is necessary (suspected insomnia) ( Soldatos et al., 2003). Respondents were then dichotomized into two groups: the insomnia group (AIS total score ≥6) and the no-insomnia group (AIS total score ≤5).

***Occupational stress***

Chronic occupational stress as perceived by the workers was assessed using the Brief Scales for Job Stress (BSJS) ( Nishikido N, Kageyama T, Kobayasi T, 2000). The BSJS is a 20-item questionnaire developed by Nishikido and colleagues, based on the job demand-control-support model similar to the Job Content Questionnaire ( Karasek et al., 1998). All participants were asked to “select the response that most closely matches your feelings about the descriptions of your current working circumstances”. Responses were rated on a 4-point scale (from 1=“disagree” to 4=“agree”), and the mean scores (range: 1.00–4.00) were calculated for six subscales: “workload,” “mental workload,” “interpersonal relationships,” “job control,” “reward from work,” and “support from colleagues and superiors.” These subscales have sufficient internal consistencies ( Nishikido N, Kageyama T, and Kobayasi T, 2000). Workload, mental workload, and interpersonal relationships were defined as job-related stress, while job control, reward from work, and support from colleagues and superiors were defined as buffers against it.

***Other covariates***

The covariates were age; sex (man, woman); smoking history (current, former, non-smoker); education (high school, university, graduate university, other school); annual house income (less than 4 million yen, more than 4 million yen and less than 8 million yen, more than 8 million yen and less than 12 million yen, more than 12 million yen); exercise habits (a few times a month, once a week, twice a week, 3 or more times a week); marriage status (unmarried, married, divorced, bereaved); presence of children (yes, no); working years (less than 1 year, more than 1 year and less than 3 years, more than 3 years and less than 5 years, more than 5 years and less than 10 years, more than 10 years); regularly go to a hospital (yes, no); residence (Tsukuba science city, Ibaraki prefecture, Tokyo-metropolitan, other); occupation (research or education, office work, technical job, other); job type (full-time, fixed-term, full-time, part-time, permanent); and type of organization (national, agency, private, corporation).

***Statistical analysis***

The AIS rates 5 points or less as no insomnia symptoms, and 6 points or more, as insomnia symptoms. A crosstabulation table was prepared for those with and without insomnia symptoms by AIS, and chi-square and unpaired t-tests were performed.

We conducted a binomial logistic regression analysis with two groups as the objective variables. We created models with childhood bullying experience and domestic violence experience as explanatory variables and additional inputs of age, gender, smoking habits, family income, education, exercise habits, marital status, presence of children, hospital visits, years of work, place of residence, and BSJS as covariates. The BSJS was entered into a logistic regression model with the mean scores of each of the subscales as quantitative variables. Multicollinearity was checked in each model. Hosmer-Lemeshow tests were conducted to examine the quality of each model. Also, Nagelkerke pseudo R-squared measures was calculated.

All statistical tests were 2-sided, and probability values below 0.05 were considered to indicate significance. We also calculated the 95% confidence intervals.

EZR version 1.40 (Saitama Medical Center, Jichi Medical University, Saitama, Japan) ( Kanda, 2013) were used for the statistical analysis. EZR is a graphical user interface for R (The R Foundation for Statistical Computing, Vienna, Austria). More precisely, it is a modified version of R commander designed to add statistical functions frequently used in biostatistics.

***Ethical considerations***

The web survey contained clear statements that participation was entirely voluntary, that it was an anonymous survey, that the privacy of the respondent would be respected, and that the data would be strictly controlled. In *The 7th Life Environment and Workplace Stress Survey*, the respondents were not told in advance that the data would be used for research purposes.

The consent of the participants was taken when The Tsukuba Science City Network conducted the study. We received permission from The Tsukuba Science City Network to use the data for our research. We published an opt-out notice on our laboratory’s homepage, stating that the data would be used for research purposes without any name on the participants’ names ( http://occup-aerospace-psy.org/content/files/7tsukukyov3R1.pdf).

From the 8th survey, we will inform respondents in advance that the collected data will be used for research purposes. This research proposal was reviewed and approved by the ethics committee of the University of Tsukuba (approval #1374). All procedures were conducted in accordance with the ethical standards of the national research committee and the Helsinki Declaration.

## Results

### Descriptive characteristics

Table 1 and Table 2 show the participants’ characteristics. The insomnia group consisted of 2997 patients (41.8%) and the non-insomnia group consisted of 4178 patients (58.2%). A significant difference was found in the number of individuals in the insomnia and non-insomnia groups between the bullying victim group and the DV victim group (chi-squared test, p<0.001). The insomnia group was also higher on all means of the BSJS subscales (unpaired t-test, all p<0.001).

**Table 1.** Characteristics.

		AIS		
		≤5 (n=4178)	≥6 (n=2997)	p value
Age	Mean (SD)	44.4 (10.5)	43.9 (10.2)	0.041 ^a^
Sex (%)	Men	2588 (61.9)	1921 (64.1)	0.066 ^b^
	Women	1590 (38.1)	1076 (35.9)	
Marriage status (%)	Single	984 (23.6)	846 (28.2)	<0.001 ^b^
	Married	3070 (73.5)	2000 (66.7)	
	Bereavement	24 ( 0.6)	27 ( 0.9)	
	Divorced	100 ( 2.4)	124 ( 4.1)	
Smoking status (%)	Current smoker	453 (10.8)	323 (10.8)	0.461 ^b^
	Former smoker	748 (17.9)	571 (19.1)	
	Non-smoker	2977 (71.3)	2103 (70.2)	
Education status (%)	Graduateschool	2039 (48.8)	1395 (46.5)	0.067 ^b^
	High school	535 (12.8)	425 (14.2)	
	Other school ^c^	468 (11.2)	376 (12.5)	
	University	1136 (27.2)	801 (26.7)	
Exercise (%)	No exercise habit	801 (19.2)	435 (14.5)	<0.001 ^b^
	Few times per month	564 (13.5)	366 (12.2)	
	One time per week	697 (16.7)	496 (16.5)	
	2 times per week	649 (15.5)	546 (18.2)	
	3 or more times per week	1467 (35.1)	1154 (38.5)	
Presence of children (%)	No	1625 (38.9)	1350 (45.0)	<0.001 ^b^
	Yes	2553 (61.1)	1647 (55.0)	
Regularly go to a hospital (%)	No	2787 (66.7)	1760 (58.7)	<0.001 ^b^
	Yes	1391 (33.3)	1237 (41.3)	
Annual income (%)	0-4 million yen	601 (14.4)	523 (17.5)	<0.001 ^b^
	4 million yen - 8 milion yen	1481 (35.4)	1104 (36.8)	
	8 million yen - 12 million yen	1420 (34.0)	971 (32.4)	
	≤12 million yen	676 (16.2)	399 (13.3)	
Woking years (%)	Less than 1 year	367 ( 8.8)	242 ( 8.1)	0.207 ^b^
	More than 1 year, less than 3 years	532 (12.7)	392 (13.1)	
	More than 3 years, less than 5 years	346 ( 8.3)	281 ( 9.4)	
	More than 5 years, less than 10 years	758 (18.1)	577 (19.3)	
	More than 10 years	2175 (52.1)	1505 (50.2)	

*Note.* AIS represnets Athens Insomnia Scale.

^a^ Unpaired t-test was used.

^b^ Chi-square test was used.

^c^ Vocational school, junior college.

**Table 2.** Characteristics.

		AIS		
		≤5 (n=4178)	≥6 (n=2997)	p value
Bullying experisnces (%)	Never experienced	1971 (47.2)	1240 (41.4)	<0.001 ^b^
	Experienced one period	1565 (37.5)	1064 (35.5)	
	Experienced two periods	477 (11.4)	491 (16.4)	
	Experienced three periods	130 (3.1)	131 (4.4)	
	Experienced four periods	35 (0.8)	71 (2.4)	
DV experiences (%)	Never experienced	3606 (86.3)	2433 (81.2)	<0.001 ^b^
	Experienced one period	305 (7.3)	267 (8.9)	
	Experienced two periods	189 (4.5)	172 (5.7)	
	Experienced three periods	50 (1.2)	81 (2.7)	
	Experienced four periods	28 (0.7)	44 (1.5)	
Workload	Mean (SD)	2.03 (0.82)	2.32 (0.93)	<0.001 ^a^
Mental workload	Mean (SD)	2.06 (0.79)	2.42 (0.91)	<0.001 ^a^
Interpersonal relationships	Mean (SD)	1.84 (0.70)	2.22 (0.85)	<0.001 ^a^
Job control	Mean (SD)	2.85 (0.73)	2.64 (0.80)	<0.001 ^a^
Support from colleagues and superiors	Mean (SD)	2.89 (0.65)	2.64 (0.72)	<0.001 ^a^
Reward from work	Mean (SD)	2.88 (0.83)	2.62 (0.88)	<0.001 ^a^

*Note.* AIS represnets Athens Insomnia Scale.

^a^ Unpaired t-test was used.

^b^ Chi-square test was used.

### Binomial logistic regression analysis

Table 3 and Table 4 show the results of a binomial logistic regression analysis. A Hosmer-Lemeshow test was performed on the model and the probability value was less than 0.05, indicating a poor fit (p<0.001). Nagelkerke pseudo R-squared measures was 0.151. Job type and occupation were excluded from the explanatory variables because multicollinearity was obtained (variance inflation factor>10).

**Table 3.** Binomial logistic regression analysis with insomnia (AIS≥6) as the dependent variable.

Variables		OR (95%CI)	p value
Bullying experiences (Ref. Never experienced)	Experienced one period	1.38 (1.19-1.60)	<0.001
	Experienced two periods	1.03 (0.79-1.34)	0.84
	Experienced three periods	1.83 (1.19-2.84)	0.0064
DV experiences (Ref. Never experienced)	Experienced one period	0.99 (0.78-1.24)	0.90
	Experienced two periods	1.82 (1.24-2.68)	0.0022
	Experienced three periods	2.06 (1.24-3.43)	0.0053
BSJS	Workload	1.15 (1.06-1.24)	<0.001
	Mental workload	1.42 (1.31-1.55)	<0.001
	Interpersonal relationships	1.38 (1.28-1.48)	<0.001
	Job control	0.92 (0.85-1.00)	0.062
	Support from colleagues and superiors	0.86 (0.78-0.94)	<0.001
	Reward from work	0.82 (0.76-0.88)	<0.001

*Note.* OR and CI represnts Odds Ratio and Confidence Interval, respectively. Ref. and BSJS represents Reference and Brief Scales for Job Stress, respectively.

**Table 4.** Binomial logistic regression analysis with insomnia (AIS≥6) as the dependent variable.

Variables		OR (95%CI)	p value
Age		1.00 (1.00-1.01)	0.64
Sex (Ref. Men)	Woman	0.93 (0.82-1.05)	0.26
Smoking (Ref. Non-smoker)	Current smoker	0.92 (0.78-1.10)	0.37
	Former smoker	1.10 (0.96-1.27)	0.16
Exercise (Ref. No exercise habit)	Few times per month	1.11 (0.96-1.28)	0.16
	One time per week	0.95 (0.82-1.10)	0.48
	2 times per week	0.85 (0.72-1.00)	0.043
	3 or more times per week	0.75 (0.64-0.87)	<0.001
Regularly go to a hospital (Ref. No)	Yes	1.32 (1.18-1.47)	<0.001
Marriage status (Ref. Single)	Married	1.05 (0.89-1.24)	0.59
	Divorced	0.67 (0.49-0.91)	0.011
	Bereavement	0.62 (0.34-1.14)	0.12
Presence of children (Ref. None)	Yes	0.90 (0.78-1.04)	0.16
Annual house income (Ref. <4 million yen)	4 million yen - 8 milion yen	1.27 (1.09-1.49)	0.0029
	8 million yen - 12 million yen	1.25 (1.05-1.49)	0.012
	≤12 million yen	1.35 (1.10-1.67)	0.0051
Education (Ref. High school)	University	0.85 (0.72-1.02)	0.075
	Other school ^a^	1.10 (0.89-1.34)	0.38
	Graduate school	0.79 (0.67-0.95)	0.01
Working years (Ref. Less than 1 year)	More than 1 year, less than 3 years	1.04 (0.84-1.30)	0.70
	More than 3 years, less than 5 years	1.17 (0.92-1.49)	0.20
	More than 5 years, less than 10 years	1.06 (0.86-1.31)	0.59
	More than 10 years	0.92 (0.74-1.13)	0.40
Residence (Ref. Other)	Tsukuba science city	0.97 (0.79-1.19)	0.77
	Ibaraki prefecture	0.93 (0.75-1.15)	0.48
	Tokyo-metropolitan	1.10 (0.81-1.51)	0.54
Type of organization (Ref. Corporation)	National	1.01 (0.84-1.21)	0.93
	Agency	1.05 (0.93-1.19)	0.40
	Private	1.08 (0.92-1.26)	0.36

*Note.* OR and CI represnts Odds Ratio and Confidence Interval, respectively. Ref. represents Reference.

^a^ Vocational school, junior college.

Regarding childhood bullying experiences, there was a trend toward insomnia in the group that experienced one period of bullying and in the group that experienced three periods of bullying compared to the group that never experienced (OR 1.38, 95%CI 1.19-1.60, OR 1.83, 95%CI 1.19-2.84, respectively). On the other hand, regarding childhood experience of domestic violence, there was a trend toward insomnia in the groups that experienced two periods and three periods compared to the group that never experienced (OR 1.82, 95%CI 1.24-2.68, OR 2.06, 95%CI 1.24-3.43, respectively). In BSJS, workload (OR 1.15, 95%CI 1.06-1.24), mental workload (OR 1.42, 95%CI 1.31-1.55), and interpersonal relationship (OR 1.38, 95%CI 1.28-1.48) were positively associated with insomnia as stress load factors, respectively. Regarding mitigating factors, Support from colleagues and superiors (OR 0.86, 95%CI 0.78-0.94) and Reward from work (OR 0.82, 95%CI 0.76-0.88) were negatively associated with insomnia, but not significantly associated with job control (OR 0.92, 95%CI 0.85-1.00).

## Discussion/conclusion

Our aim was to examine whether childhood experiences of bullying and DV were associated with insomnia among workers in adulthood. Childhood experiences of bullying and DV tended to cause statistically significant insomnia in a model that also adjusted for age, gender, lifestyle, income, and occupational factors. As for the experience of DV, the higher the time of experience, the higher the odds ratio of insomnia. Ours is the first large cross-sectional study in Japan to examine the influence of bullying and DV experiences on sleep.

The detail mechanism by which childhood experiences of domestic violence/bullying lead to insomnia in adulthood is unclear, but two hypotheses have been proposed ( Kajeepeta et al., 2015). One possibility is that ACE increases corticotropin-releasing hormone (CRH) reactivity, which may later affect sleep quality; elevated CRH, and subsequent enhancement of the hypothalamic-pituitary-adrenal (HPA) axis, is associated with reduced sleep. However, studies on the relationship between ACE and cortisol are inconclusive. HPA axis responses have been suggested to be under genetic and epigenetic influence, and adverse childhood experiences may also influence HPA axis activity in adulthood ( Morris et al., 2019).

Employees with ACEs are more likely than those without ACEs to seek or need health care services related to the chronic physical and mental health conditions associated with this (Arbesman & Logsdon, 2011). Therefore, occupational health nurses and occupational health physicians are likely to meet with individuals with ACEs. The importance of trauma informed care (TIC) has recently been reported as a care for ACEs ( Huang et al., 2014). In employees who complain of insomnia, occupational health workers may also identify and address the risk of ACEs. TIC is a framework for acquiring trauma knowledge and responses and supporting appropriate responses for traumatized individuals. TIC is offered in the community as well as in medical institutions ( Mahon, 2022) and can be applied in occupational health settings ( Rosemberg et al., 2017), as cognitive behavioral therapy that includes TIC has been reported to improve insomnia in PTSD ( Carlson et al., 2022), TIC could be practiced in occupational health settings to improve insomnia in workers with ACEs.

This study has the following limitations. First, it was a cross-sectional study, so a clear causal relationship was not determined. Second, the response rate of this survey was low—37.2%. There have a been study those voluntary respondents reported better physical and mental health than mandatory respondents. This survey relies on voluntary responses, which may have bias in the results. Because the response rate in this survey was low, ways to increase the response rate in the future must be devised. Moreover, we surveyed mainly past experiences, so remembering past actions and emotions was eventually reduced. Third, to investigate the experiences of bullying/DV, the previously validated Bullying and Friendship Interview Schedule ( Wolke et al., 2012)/Adverse Childhood Experiences Questionnaire ( Felitti MD et al., 1998) may have yielded more accurate data. Fourth, we used a self-report questionnaire called the AIS to investigate insomnia. Self-reported formulas are known to have recall bias. For example, self-reported sleep duration overestimates objective measures of sleep such as those by polysomnography and actigraphy ( Jackson et al., 2018). Moreover, in this study, insomnia was not diagnosed by a doctor. Finally, unmeasured confounding factors (such as drinking habits and history of mental illness) were not eliminated.

In conclusion, we showed that workers with childhood experience of bullying or DV tend to have insomnia. In the future, objective sleep time and sleep efficiency should be evaluated using an activity meter and other methods to verify the effects of bullying and DV experiences.

## Data Availability

Zenodo: Relationships Between Childhood Bullying/Domestic Violence Experience and Insomnia among Employees in Japan, https://doi.org/10.5281/zenodo.7487997 ( Muroi *et al., *2022). This project contains the following underlying data:
-
Tsukuba_Cross_Sectional.sav Zenodo: Relationships Between Childhood Bullying/Domestic Violence Experience and Insomnia among Employees in Japan, https://doi.org/10.5281/zenodo.7487997 ( Muroi *et al., *2022). This project contains the following extended data:
- Original Questionnaire (English).pdf - Original Questionnaire (Japanese).pdf Data are available under the terms of the Creative Commons Attribution 4.0 International license (CC-BY 4.0).
