# Supplementary material for: Relationships Between Childhood Bullying/Domestic Violence Experience and Insomnia among Employees in Japan
Source: F1000Res. 2023 Jul 14;12:103. Originally published 2023 Jan 27. [Version 2] doi: 10.12688/f1000research.129340.2 (PMC10293797; doi:10.12688/f1000research.129340.2)
Supplement: Peer review history

# Peer review: Reviewer response for version 1

**DOI:** 10.5256/f1000research.142020.r174528

**Published:** 2023-06-26

**Authors:** Farizah Mohd Hairi

**Affiliations:** 1 Centre for Epidemiology and Evidence-Based Practice, Department of Social and Preventive Medicine, Faculty of Medicine, University of Malaya, Kuala Lumpur, Malaysia

Abstract:

 This study investigates the association between childhood bullying/domestic violence experience and insomnia among employees in Japan. The research aims to explore the potential long-term effects of early-life adversity on sleep patterns and overall well-being. The study utilizes a cross-sectional design and collects data through self-report questionnaires. The findings suggest a significant positive relationship between childhood bullying/domestic violence experience and insomnia symptoms. The implications of these results highlight the importance of addressing past traumatic experiences when considering insomnia prevention and intervention strategies in the workplace.

Introduction:

 The introduction provides a comprehensive overview of the research topic, highlighting the prevalence of childhood bullying and domestic violence in Japan and its potential impact on individuals' long-term health. It discusses the existing literature on the association between early-life adversity and sleep disturbances, emphasizing the need for further investigation in the context of the Japanese workforce. The section concludes by outlining the research objectives and hypotheses.

Methods:

 This section outlines the study design, participants, and data collection procedures. It describes the sample selection process, including inclusion and exclusion criteria. The measurement tools used to assess childhood bullying, domestic violence experience, and insomnia symptoms are described in detail, along with their psychometric properties. Ethical considerations and data analysis methods are also presented.

Results:

 The results section presents the findings of the study, organized in a clear and concise manner. It provides descriptive statistics of the sample characteristics, prevalence rates of childhood bullying and domestic violence experience, and the distribution of insomnia symptoms among participants. The main analysis examines the relationship between childhood bullying/domestic violence experience and insomnia, utilizing appropriate statistical tests. The section includes effect sizes and confidence intervals where applicable.

Discussion:

 The discussion interprets the study findings in light of existing literature. It explores the possible mechanisms underlying the relationship between childhood adversity and insomnia symptoms, considering psychological, physiological, and socio-environmental factors. The implications of the findings for occupational health and interventions in the workplace are discussed, emphasizing the importance of addressing early-life trauma in promoting healthy sleep patterns among employees. The limitations of the study are acknowledged, and suggestions for future research are provided.

Conclusion:

 The conclusion provides a concise summary of the research findings and their implications. It emphasizes the significance of the study in shedding light on the relationship between childhood bullying/domestic violence experience and insomnia among employees in Japan. The section concludes by reiterating the importance of addressing early-life trauma and implementing appropriate interventions to promote healthy sleep and overall well-being in the workplace.

 Overall, the manuscript effectively investigates the relationships between childhood bullying/domestic violence experience and insomnia among employees in Japan. It contributes to the existing literature by highlighting the long-term effects of early-life adversity on sleep patterns and providing insights into potential intervention strategies. The study's rigorous methodology, clear presentation of results, and comprehensive discussion enhance the scientific value of the research. Suggestions for further improvements could include a larger and more diverse sample, longitudinal study design, and consideration of other potential confounding variables.

Is the work clearly and accurately presented and does it cite the current literature?

Yes

If applicable, is the statistical analysis and its interpretation appropriate?

Yes

Are all the source data underlying the results available to ensure full reproducibility?

Yes

Is the study design appropriate and is the work technically sound?

Yes

Are the conclusions drawn adequately supported by the results?

Yes

Are sufficient details of methods and analysis provided to allow replication by others?

Yes

Reviewer Expertise:

social epidemiology, care of older persons

I confirm that I have read this submission and believe that I have an appropriate level of expertise to confirm that it is of an acceptable scientific standard.

# Peer review: Reviewer response for version 1

**DOI:** 10.5256/f1000research.142020.r163521

**Published:** 2023-03-22

**Authors:** Gerardo Ochoa-Meza

**Affiliations:** 1 Departamento de Ciencias Sociales, Universidad Autónoma de Ciudad Juárez, Ciudad Juárez, Mexico

I have a suggestion about the summary. The article can improve its scientific soundness if a clear, brief and concise conclusion is added in the abstract. The conclusion must be written according to what is mentioned in the Discussion/conclusions of this article.

 In general, it is an excellent article despite the limitations indicated in the article. I consider that it is a contribution to continue advancing in this line of research.

Is the work clearly and accurately presented and does it cite the current literature?

Yes

If applicable, is the statistical analysis and its interpretation appropriate?

Yes

Are all the source data underlying the results available to ensure full reproducibility?

Yes

Is the study design appropriate and is the work technically sound?

Yes

Are the conclusions drawn adequately supported by the results?

Yes

Are sufficient details of methods and analysis provided to allow replication by others?

Yes

Reviewer Expertise:

NA

I confirm that I have read this submission and believe that I have an appropriate level of expertise to confirm that it is of an acceptable scientific standard.
